# The spatio-temporal distribution of alkaline phosphatase activity and *phoD* gene abundance and diversity in sediment of Sancha Lake

**DOI:** 10.1038/s41598-023-29983-1

**Published:** 2023-02-22

**Authors:** Yong Li, Xintao Yu, Huan Liu, Zhilian Gong

**Affiliations:** 1 Faculty of Geosciences and Environmental Engineering, Southwest Jiaotong University, Chengdu, 610059 China; 2 School of Food and Biological Engineering, Xihua University, Chengdu, 610039 China

**Keywords:** Ecology, Environmental sciences

## Abstract

The bacterial *phoD* gene encoding alkaline phosphatase (ALP) plays an important role in the release of soluble reactive phosphorus (SRP) from organic phosphorus in ecosystems. However, *phoD* gene diversity and abundance in ecosystems is poorly understood. In the present study, we sampled the surface sediments and the overlying water of Sancha Lake at 9 different sampling sites, a typical eutrophic sub-deep freshwater lake in China, in April 15 (spring) and November 3 (autumn), 2017. High-throughput sequencing and qPCR were performed to analyze the diversity and abundance of the bacterial *phoD* gene in the sediments. We further discussed the relationships between the diversity and abundance of the *phoD* gene and environmental factors and ALP activity. A total of 881,717 valid sequences were obtained from 18 samples and were classified into 41 genera, 31 families, 23 orders, 12 classes, and 9 phyla and grouped into 477 OTUs. The dominant phyla were Proteobacteria and Actinobacteria. The phylogenetic tree based on the sequences of the *phoD* gene was plotted and composed of three branches. The genetic sequences were aligned predominantly with genera *Pseudomonas*, *Streptomyces*, *Cupriavidus*, and *Paludisphaer*. The *phoD*-harboring bacterial community structure showed a significant difference in spring and autumn, but no apparent spatial heterogeneity. The *phoD* gene abundances at different sampling points were significantly higher in autumn than in spring. In autumn and spring, the *phoD* gene abundance was significantly higher in the tail of lake and where cage culture used to be intensive. pH value, dissolved oxygen (DO), total organic carbon (TOC), ALP, and phosphorus were important environmental factors affecting the diversity of the *phoD* gene and the *phoD*-harboring bacterial community structure. Changes in *phoD*-harboring bacterial community structure, *phoD* gene abundance, and ALP activity were negatively correlated with SRP in overlying water. Our study indicated *phoD*-harboring bacteria in the sediments of Sancha Lake with the characteristics of high diversity and significant spatial and temporal heterogeneity in abundance and community structure, which played a important role in the release of SRP.

## Introduction

Eutrophication was caused by high concentration of nutrients such as nitrogen and phosphorus. Phosphorus was usually the limiting factor of lake eutrophication^1^. With exogenous phosphorus properly controlled, phosphorus released from lake sediments became the main source of water eutrophication^2^. Studies on the release of endogeneous phosphorus from sediments have primarily focused on the impact of environmental physical and chemical factors on morphological transformation and release amount^3–6^. However, there were few studies on microbial taxa involved in phosphorus release. Phosphorus in sediments would be released into the overlying water through physical, chemical and biological interactions between sediment and overlying water^7^. Microorganisms played a crucial role in the phosphorus cycle at the sediment–water interface and the endogenous phosphorus release from sediments^8^. According to the method for phosphorus fraction in sediment developed under the frame of Standard Measurements and Testing (SMT) Program of the European Commission, the phosphorus forms in the sediments were classified into total phosphorus (TP), organic phosphorus (OP), inorganic phosphorus (IP), NaOH-P and HCl-P. Among them, OP was an important part of the phosphorus balance in the lake ecosystem, and also an important phosphorus source released from the sediments of eutrophic lakes. OP in sediments was mainly released through mineralization and decomposition of Alkaline phosphatase (ALP) secreted by microorganisms. ALP is responsible for dephosphorylation of organic phosphorus compounds by breaking the phosphodiester linkage to remove phosphate groups, and thereby forming orthophosphates. Algae utilized orthophosphates and proliferated massively, which resulted in water eutrophication. ALP activity and stability were mainly affected by pH, T, DO, PO_4_^3−^ and other environmental factors^9^.

ALP is an important enzyme that plays an important role in organic phosphorous hydrolysis^10^. Three genes mainly encode for ALP, namely, *phoA*, *phoX*, and *phoD*^11^. It is generally believed that the genetic diversity and abundance of the *phoD* gene are higher than those of the *phoA* and *phoX* genes in the soil and marine ecosystems^12,13^. An increasing number of researchers have been devoted to the study of the *phoD* gene, which can be used as a molecular marker. High-throughput sequencing is usually employed to study the *phoD* gene diversity, abundance and distribution in soil ecosystems^14,15^. On this basis, we can further discuss the influence of fertilization, soil pH, salinity, and other factors on *phoD* gene abundance and microbial community structure^13,16,17^. These results indicated that the expression of *phoD* gene was restricted by phosphorus content and salinity stress. Fertilization would increase the *phoD* gene abundance and change the *phoD*-harboring bacterial community structure. Relevant researches have also shown that the *phoD* gene exists abundantly in the ocean surface and plays an important role in the degradation of organic phosphorus in oceans^12,18^.

In the past, *phoD* gene in soil and marine ecosystems was reported in most studies, but *phoD* gene in the freshwater lake ecosystems was seldom studied. The studies of suspended particles *phoD* alkaline phosphatase gene diversity, effects of cyanobacterial growth and decline on the *phoD*-harboring bacterial community structure, and changes of *phoD* gene community in sediments in different seasons have been conducted in the shallow freshwater lake systems^19–21^. These results indicated that the diversity and abundance of *phoD* genes were high in the freshwater lake ecosystems. PH, T, DO, SRP, OP, TP and TN/TP were the main environmental factors affecting the diversity and abundance of *phoD* genes^2^. ALP played an important role in the process of eutrophication of water bodies.

No researches have been conducted on the diversity of *phoD* gene and its relationship with eutrophication in the sediments from sub-deep freshwater lake (between deep and shallow lake) systems. Investigating the diversity of the *phoD* gene in the sediments of eutrophic lakes and the microbial community structure based on the *phoD* gene can help identify the microbial communities that have an important influence on phosphorus release. Microbial mineralization of OP is an important component of phosphorus cycle in sub-deep freshwater lake ecosystems. The research on *phoD* gene is helpful to reveal microbial driving mechanism of OP mineralization in eutrophic sub-deep freshwater lake area. Besides, *phoD*-harboring microbial communities may make varying contributions to phosphate solubilization under different environmental conditions. For these reasons, it is highly necessary to study the intrinsic relationships between *phoD*-harboring microbial communities and environmental factors. Sancha Lake is a sub-deep freshwater lake featured by endogenous pollution and eutrophication^22^. The release of endogenous phosphorus from the sediments of Sancha Lake is the main cause of water eutrophication^23^. We have focused on the response of *phoD*-harboring microbial communities in the sediments of Sancha Lake. Here, we hypothesized a high diversity of the *phoD* gene encoding ALP in the sediments of Sancha Lake and that the *phoD* gene abundance and the *phoD*-harboring microbial communities vary seasonally and spatially as a response to environmental factors. In addition, *phoD*-harboring microbial communities make important contributions to soluble reactive phosphorus (SRP).

We performed total DNA extraction and qPCR and high-throughput sequencing of the bacterial *phoD* gene in the sediments of Sancha Lake. On this basis, we analyzed the correlations between the diversity and spatial–temporal distribution of the *phoD* gene in sediments vs. dissolved oxygen (DO), temperature (T), pH, total organic carbon (TOC), total nitrogen (TN), and phosphorus.

## Materials and method

### Description of the sampling sites and sediment collection

**Figure 1:**
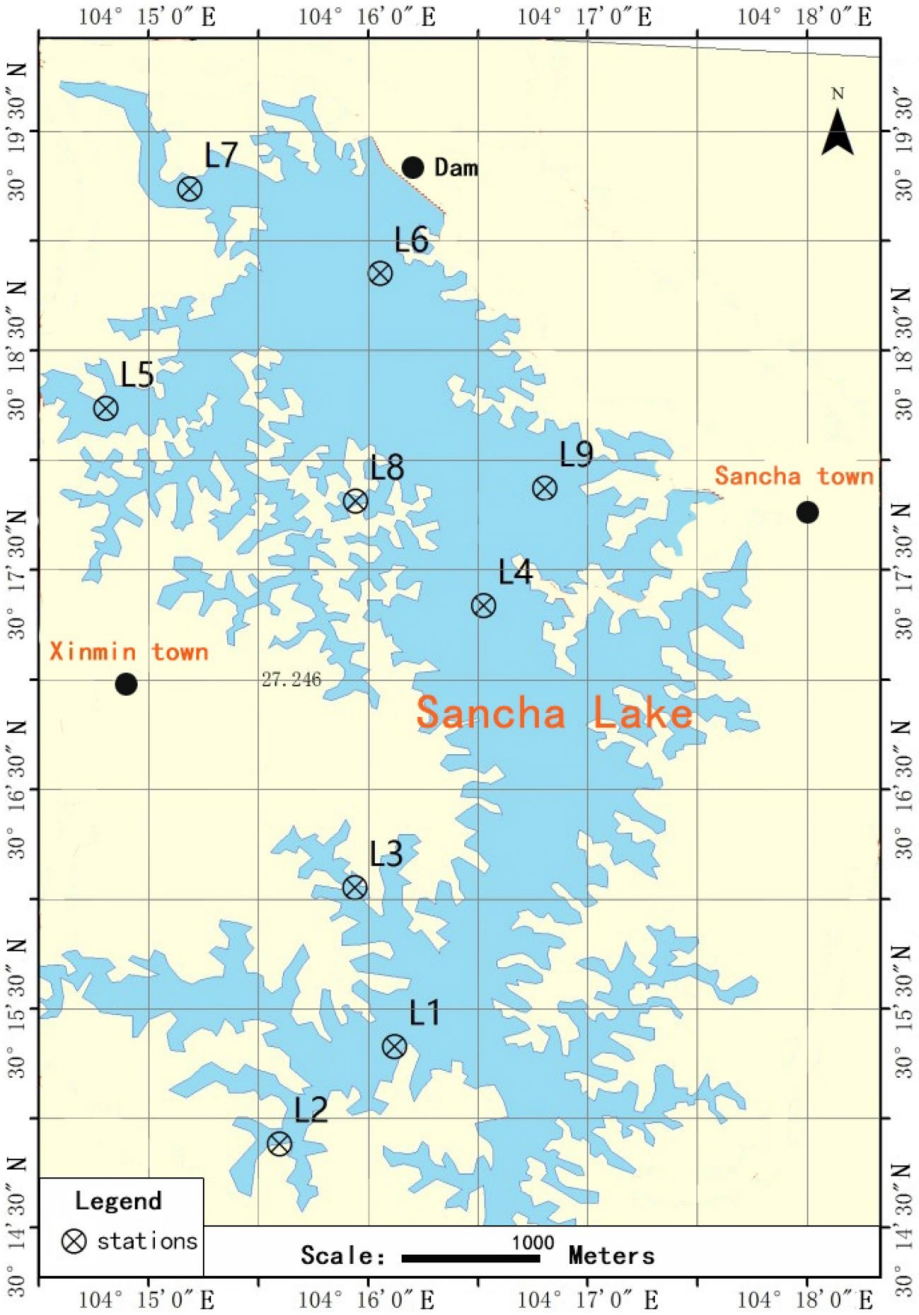
Sampling sites in the Sancha Lake.

Sancha Lake is situated in Sichuan Tianfu New Area (E 104°11′16″ ~ E 104°17′16ʺ, N 30°13′08″ ~ N 30°19′56″), in the eastern suburb of Chengdu and upstream of Jiangxi River, a tributary of Tuojiang River belonging to the Yangtze River System. The average water depth of Sancha Lake is 8.3 m and the maximum water depth 32.5 m. The study area has a subtropical humid monsoon climate, with an average temperature of 15.2–16.9 °C and an average precipitation of 786.5 mm. The catchment area above the dam site is 161.25 km^2^. The water source of Sancha Lake was mainly from the Min River, accounting for about 80% of the total water volume of the reservoir. The rest came from rainfall and two creeks. Spring was the period of irrigation and drainage, and autumn was the period of water diversion. Sancha Lake is not only an important water source area in Tianfu New Area, but also performs various functions, including maintaining biodiversity, storing water for agricultural irrigation, and regulating surface runoff and local climate. COD_Cr_ and BOD_5_ in the inflow water have been decreasing according to the water quality monitoring over the years. By contrast, TP in the lake water has been rising. Correspondingly, Chla also increases, while transparency has been decreasing over the years, resulting in an increasing severity of eutrophication^24^. Based on the features of sediment distribution and eutrophication status in Sancha Lake, 9 sampling points were selected, as shown in Fig. 1^25^. The latitude and longitude of the sampling points were determined using a GPS unit. The sampling points L1 and L4 were located where concentrated area of fenced aquaculture used to be intensively carried out. The sampling points L2 and L3 were located in the tailwater area. The sampling point L9 was close to regions with intense human activities. The sampling points L5, L8 and L6 (dam) were closed to where cage culture used to be highly intense. The sampling point L7 was located in the main inflow water area. At the nine chosen sampling points as shown in Fig. 1, the surface sediments (0–10 cm) from the lake bottom were collected respectively with a Peterson grab and then sealed in polyethylene bags in April 15, 2017 (Spring) and November 3, 2017 (Autumn). Three samples were collected for each site and mixed together as the representative sample for this site. The samples were placed in polyethylene bags and sealed. They were frozen in ice and immediately taken back to the laboratory. One part of the sample was stored in the fridge at 4 °C for physicochemical analysis (within 24 h). The remaining part was stored in the fridge at − 80 °C for DNA extraction. Besides, at each sampling point, an airtight water sampler was used to collect water overlying the sediments for analyzing water environmental indicators.

### Determination of physical and chemical factors in the sediments and overlying water

The sequential P extraction tests in sediments were conducted by SMT^26,27^. Sequential P extraction in sediments by SMT Method includes TP, OP, NaOH-P and HCl-P in sediments. SMT method has three independent steps: 3.5 mol L^−1^ HCl extracts TP, 1 mol L^−1^ HCl extracts IP and OP, and 1 mol L^−1^ NaOH extracts NaOH-P and HCl-P. TP, OP, NaOH-P and HCl-P in sediments were determined using the ammonium molybdate spectrophotometric method^28^. TOC in sediments was determined according to the standard GB/T 19145-2003 released by the General Administration of Quality Supervision, Inspection and Quarantine of the People's Republic of China. TN in sediments was determined using alkaline potassium persulfate digestion UV spectrophotometric method^25^. The pH values of freshly collected sediments were determined using the glass electrode method after extraction with the ratio of sediments to distilled water being 1:5. The overlying water samples were passed through a 0.45 μm membrane filter. The filtrate was digested with potassium sulfate, and dissolved total phosphorus (DTP) was determined using ammonium molybdate spectrophotometric method. The overlying water samples were passed through a 0.45 μm membrane filter. The filtrate was measured for SRP using ammonium molybdate spectrophotometric method. Dissolved organic phosphorus (DOP) was DTP minus SRP^29^. T was measured with the HI991301 portable multiparameter meter; DO was measured with the HQ30D Portable Dissolved Oxygen Meter.

### Sediment ALP activity assay

The ALP activity was measured using Anupama's method^30^, specifically as follows: About 0.5 g of freshly collected sediments was weighed and placed in the disinfected reaction tube. Into the tube 10 mL of 0.5 mol L^−1^ Tris–HCl buffer with a pH value of 8.6 was added and mixed well by shaking, followed by ultrasonic treatment for 45 s. Into the mixture 1 mL of 10 mmol L^−1^ p-nitrophenyl Phosphate was added as substrate, mixed well and placed in the 37 °C water bath for 1 h. The reaction was terminated by adding 2 m of 1 mmol L^−1^ NaOH solution. The solution was then subjected to refrigerated centrifugation at 5000 rpm at 10 min, and the supernatant was collected. The absorbance was measured using a spectrophotometer at 410 nm and converted to activity intensity per gram (dry weight) of sediments. A standard curve was plotted for varying contents of p-nitrophenol (PNP). Each sample had three replicates. ALP activity was expressed as μmol PNP produced by each gram (dry weight) of sediments per hour.

### DNA extraction, PCR and Illumina Miseq sequencing

Total DNA was extracted from the samples according to the instruction provided with the E.Z.N.A.® soil kit (Omega Bio-tek, Norcross, GA, US). DNA concentration and purity were determined using a Nanodrop 2000 spectrophotometer. The DNA quality was evaluated by 1% agarose gel electrophoresis.

The *phoD* gene abundance was quantified using the TIB8600 fluorescence qPCR analyzer (Triplex International Biosciences (China) Co., Ltd.). The primers used to amplify the *phoD* gene were ALPS-F730 (5′-CAGTGGGACGACCACGAGG T-3′) and ALPS-R1101 (5′-GAGGCCGATCGGCATGTCG-3′)^17,31,32^. Ten-fold dilution was performed for all DNA samples. About 10 ng of DNA was used as template for each qPCR reaction. Each sample had three replicates, and a negative control was set up. The qPCR reaction system (20 μL) consisted of the following: 9 μL 2 × SYBR real-time PCR premixture (BioTeke Corporation, Beijing, China), 0.5 μL upstream and downstream primers (10 µM), and 10 μL of diluted DNA template. The concentration of different mixed reagents in the qPCR reaction system is initial. qPCR consisted of the following steps: Predenaturation at 95 °C for 5 min, 40 cycles (denaturation at 95 °C for 15 s, annealing at 60 °C /55 °C for 30 s, extension at 72 °C for 30 s); final extension at 72 °C for 1 min. The plasmid standard harboring the target gene was constructed with the pMD18-T Vector (TaKaRa, Japan) and sequenced for verification. Standard curves were generated using tenfold serial dilutions of the plasmids. The range of *phoD* gene copy numbers is 6.87 × 10^2^ ~ 6.87 × 10^8^ from the different standard curves. The number of *phoD* gene copies was calculated by measuring the concentration of the plasmid and the number of base pairs. Amplification efficiencies ranged from 94 to 98%, and R^2^ value of 0.9932 for *phoD* gene.

The communities of *phoD*-harboring bacteria were assessed using the Illumina Miseq 300 bp paired-end sequencing platform. The variable barcodes were designed and respectively linked to the 5' end of primer pairs (ALPS-F730/ALPS-S1101) to allow distinguishing of the sequences of each sample. DNA thus obtained was used as template for *phoD* gene amplification on ABI Applied Biosystem 2720 Thermal Cycler (Applied Biosystems, Foster City, CA, USA). Amplification of each sample was done in triplicate, with 20 μL reactions under the same conditions as the qPCR experiment. The PCR amplification system consisted of the following (20 µL): 2 μL Ex Taq buffer (10 ×, 0.2 μL Ex Taq (5 U·μL^−1^), 1.6 μL dNTP Mix (2.5 mM), 1.0 μL Forward primer (5 µM), 1.0 μL Forward primer (5 µM), 0.5 μL template DNA, 13.7 μL ddH_2_O. The concentration of different mixed reagents in the PCR amplification system is initial. The PCR product was recovered using the 2% agarose gel and purified using the AxyPrep DNA Gel Extraction Kit (Axygen Biosciences, Union City, CA, USA). After assessing quantity and quality of the purified PCR products using a QuantiFluor TM-ST (Promega, USA), the three purified PCR products per sample were pooled as one PCR amplicon. Finally, the PCR amplicons were pooled in an equimolar con-centration for Illumina Miseq sequencing at Personal Biotechnology Co., Ltd. (Shanghai, China). All sequences were deposited in the NCBI Sequence Read Archive (SRA) database (BioProject number PRJNA623151).

### Bioinformatics of Illumina Miseq sequencing

Quality control of raw sequences was implemented by Trimmomatic. The sequences were merged using the FLASH software (version 2.7, http://ccb.jhu.edu/software/FLASH/, Center for Bioinformatics and Computational Biology, Iowa City, IA, USA). The sequences were clustered into OTUs at the 80% similarity threshold using the UPARSE software (version 7.1, http://drive5.com/uparse/, Edgar, R.C., Tiburon, CA, USA)^30^. Simple sequences and chimeras were excluded during the clustering process. OTUs with abundance values lower than 0.005% of the total reads of all samples were removed^33^.

RDP classifier (http://rdp.cme.msu.edu/) was used for microbial species classification and annotation for the most abundant sequence within each OTU. *phoD* gene sequences were aligned with the NCBI database (http://www.ncbi.nlm.nih.gov/), with the threshold set to 70%. Statistics were done and plots were drawn using the R-package based on the taxonomic information obtained for each OTU. Representative sequences for the top 15 most abundant OTUs were chosen. Next, the phylogenetic tree was plotted based on the representative sequences for each OTU using the maximum likelihood method and FastTree software (version 2.1.3 http://www.microbesonline.org/fasttree/)^34^.

Based on the OTU clustering results, QIIME software (version 2.0, http://qiime.org/, Rob Knight Lab, Boulder, CO, USA) was implemented to estimate the abundance-based coverage estimators (ACE), Chao1 index, Simpson index, and Shannon–Wiener index for each sample. We further examined the similarity of the structure of *phoD*-harboring bacterial communities across the samples. Principal component analysis (PCA) was conducted for microbial community structure data using QIIME^35^. The samples were ranked, and the diversity and spatial and temporal heterogeneity in the composition of *phoD*-harboring bacterial communities in sediment samples were observed. The *phoD* gene sequences were deposited in the NCBI Sequence Read Archive (SRA) database under accession number PRJNA623151.

### Statistical analysis

Variance inflation factor (VIF) was used to screen for known environmental factors that had little interaction with each other^29^. Adonis test and Metastats were implemented using the Vegan package in R to assess the significance of intragroup and intergroup differences^36^. Pearson's correlation coefficients were calculated in SPSS (version 20.0, IBM, Armonk, NY, USA) to estimate the intensity of correlations between *phoD* gene abundance, microbial biodiversity, and environmental factors. Vegan package in R was implemented to conduct redundancy analysis (RDA) between the spatial distribution features of *phoD*-harboring bacterial communities in sediments and environmental factors^37^. The significance of differences between the samples was estimated by analysis of variance (ANOVA). The significance level was set to p = 0.05, and the level of extreme significance was p = 0.01.

## Result and analysis

### Physical and chemical properties of the sediments and overlying water

The results of physicochemical factor determination in sediments and overlying water in spring and autumn are shown in Table 1. Since the Sancha Lake is a stratified reservoir, the temperature in the water overlying the sediments ranged from 11.6 ~ 17.0 °C, and did not vary significantly in spring and autumn. The value of DO ranged from 4.20 to 10.87 mg L^−1^, higher in spring than in autumn for all locations. Besides, DO was higher at the sampling point L7 in the inflow water area and at the sampling points L2 and L3 in the shallow water. The sampling point L6 in the deep water section of the dam was the lowest. At this sampling point, the value of SRP ranged from 0.006 to 0.086 mg L^−1^, higher in spring than in autumn and was higher in the inflow water area and in the arm of lake. By contrast, SRP was lower in the lake center. The value of DOP ranged from 0.001 to 0.008 mg L^−1^, lower in spring than in autumn and was higher in the tail and arm of lake than in other parts of the lake. The pH value of sediments ranged from 6.13 to 8.32, and did not vary significantly at different sampling points. Nevertheless, it was much higher in autumn than in spring. The TP value in sediments ranged from 0.27 to 2.46 mg g^−1^, higher in autumn than in spring, and it varied significantly across the sampling points in the lake area. TP was the highest where cage culture used to be intensive (L5, L6 and L8). It was the second highest where concentrated area of fenced aquaculture used to be intensive (L1 and L4), followed by the value in the region where human activities used to be intensive (L9). TP was the lowest in the inflow water area (L7) and the tailwater area (L2 and L3). These results might be explained by the practice of dropping in fish food for cage culture and the associated human activities. As indicated by TP, the water was moderately polluted according to US EPA's standard. The OP value of sediments ranged from 0.13 to 0.77 mg g^−1^. The TOC value of sediments ranged from 13.8 to 28.9 mg g^−1^. The seasonal variation and spatial distribution features of OP and TOC were consistent with those of TP. NaOH-P, HCl-P and TN did not vary significantly across the sampling points. However, these indicators were slightly higher in autumn than in spring. The ALP activity of sediments ranged from 0.22 to 2.42 μmol g^−1^ h^−1^, significantly higher in autumn than in spring. It was also higher in the tailwater area and the arm of lake where cage culture used to be intensive, but lower in the inflow area and deep water area in the dam.

**Table 1:** Physicochemical properties of the sediments and overlying water in spring and autumn.

Parameters	Season	L1	L2	L3	L4	L5	L6	L7	L8	L9
pH	Spring	6.38 ± 0.21	6.30 ± 0.25	6.20 ± 0.09	6.64 ± 0.11	6.56 ± 0.06	6.42 ± 0.15	6.13 ± 0.058	6.39 ± 0.05	6.25 ± 0.015
	Autumn	8.07 ± 0.06	7.42 ± 0.06	7.43 ± 0.24	8.32 ± 0.10	7.66 ± 0.30	7.55 ± 0.04	7.72 ± 0.055	8.20 ± 0.05	7.62 ± 0.026
DO (mg L^−1^)	Spring	9.70 ± 0.1	10.13 ± 0.15	9.67 ± 0.58	8.67 ± 0.21	9.30 ± 0.10	8.30 ± 0.10	10.87 ± 0.5	8.87 ± 0.4	8.50 ± 0.1
	Autumn	4.20 ± 0.1	5.63 ± 0.25	5.87 ± 0.21	4.93 ± 0.15	5.50 ± 0.20	4.27 ± 0.20	5.50 ± 0.2	5.60 ± 0.3	4.90 ± 0.3
T (℃)	Spring	11.90 ± 1.0	15.93 ± 0.95	13.27 ± 0.90	14.0 ± 1.0	12.63 ± 0.55	12.73 ± 0.75	14.3 ± 0.3	11.6 ± 0.4	12.6 ± 0.5
	Autumn	14.10 ± 0.2	17.0 ± 0.40	15.90 ± 0.10	15.0 ± 1.0	14.40 ± 0.40	14.20 ± 0.20	16.8 ± 0.2	13.2 ± 0.3	14.1 ± 0.2
DOP (mg L^−1^)	Spring	0.003 ± 0.001	0.006 ± 0.001	0.004 ± 0.001	0.002 ± 0.0006	0.002 ± 0.001	0.003 ± 0.001	0.001 ± 0.0006	0.002 ± 0.001	0.002 ± 0.0006
	Autumn	0.005 ± 0.001	0.008 ± 0.001	0.007 ± 0.001	0.003 ± 0.001	0.006 ± 0.0006	0.004 ± 0.0006	0.005 ± 0.0006	0.006 ± 0.001	0.011 ± 0.001
SRP (mg L^−1^)	Spring	0.05 ± 0.0002	0.03 ± 0.0001	0.01 ± 0.0002	0.042 ± 0.002	0.037 ± 0.0008	0.052 ± 0.0004	0.086 ± 0.0002	0.016 ± 0.0006	0.046 ± 0.0006
	Autumn	0.01 ± 0.0010	0.006 ± 0.001	0.009 ± 0.0006	0.008 ± 0.0006	0.052 ± 0.004	0.026 ± 0.002	0.006 ± 0.001	0.011 ± 0.001	0.01 ± 0.0060
TP (mg g^−1^)	Spring	0.58 ± 0.021	0.27 ± 0.002	0.60 ± 0.006	1.99 ± 0.09	1.75 ± 0.002	1.81 ± 0.01	0.53 ± 0.017	2.32 ± 0.01	1.03 ± 0.0017
	Autumn	0.86 ± 0.064	0.53 ± 0.099	0.82 ± 0.02	2.06 ± 0.06	2.42 ± 0.032	1.93 ± 0.04	0.71 ± 0.004	2.46 ± 0.03	1.19 ± 0.085
OP (mg g^−1^)	Spring	0.18 ± 0.031	0.13 ± 0.01	0.18 ± 0.008	0.63 ± 0.049	0.54 ± 0.01	0.32 ± 0.002	0.15 ± 0.002	0.67 ± 0.003	0.33 ± 0.0049
	Autumn	0.49 ± 0.002	0.20 ± 0.002	0.23 ± 0.002	0.65 ± 0.001	0.73 ± 0.003	0.58 ± 0.005	0.41 ± 0.002	0.77 ± 0.005	0.38 ± 0.002
HCl-P (mg g^−1^)	Spring	0.41 ± 0.002	0.21 ± 0.002	0.43 ± 0.004	1.11 ± 0.015	0.97 ± 0.009	0.99 ± 0.036	0.26 ± 0.004	1.16 ± 0.004	2.38 ± 3.14
	Autumn	0.35 ± 0.006	0.337 ± 0.007	0.43 ± 0.001	1.15 ± 0.002	1.32 ± 0.02	1.06 ± 0.02	0.33 ± 0.002	1.36 ± 0.003	0.66 ± 0.004
NaOH-P (mg g^−1^)	Spring	0.15 ± 0.001	0.14 ± 0.002	0.19 ± 0.001	0.27 ± 0.030	0.25 ± 0.01	0.23 ± 0.002	0.13 ± 0.002	0.29 ± 0.004	0.155 ± 0.001
	Autumn	0.25 ± 0.002	0.15 ± 0.010	0.24 ± 0.010	0.48 ± 0.003	0.53 ± 0.01	0.45 ± 0.001	0.24 ± 0.001	0.56 ± 0.001	0.25 ± 0.002
TOC (mg g^−1^)	Spring	20.6 ± 0.1	13.8 ± 0.1	18.0 ± 0.6	24.3 ± 0.1	25.5 ± 0.2	22.5 ± 0.1	18.4 ± 0.1	28.9 ± 0.1	19.7 ± 0.1
	Autumn	24.5 ± 0.1	18.3 ± 0.1	17.2 ± 0.1	25.9 ± 0.1	24.5 ± 0.1	23.4 ± 0.1	19.3 ± 0.1	28.5 ± 0.1	20.3 ± 0.1
TN (mg g^−1^)	Spring	2.2 ± 0.02	1.32 ± 0.02	1.4 ± 0.03	2.13 ± 0.01	2.3 ± 0.02	1.89 ± 0.01	1.48 ± 0.02	2.5 ± 0.02	2.10 ± 0.02
	Autumn	2.1 ± 0.02	1.25 ± 0.01	1.9 ± 0.03	2.53 ± 0.01	2.6 ± 0.03	1.68 ± 0.04	1.31 ± 0.01	2.4 ± 0.05	1.98 ± 0.02
ALP (μmol g^−1^ h^−1^)	Spring	0.76 ± 0.02	0.26 ± 0.01	0.55 ± 0.01	0.79 ± 0.01	0.55 ± 0.01	0.22 ± 0.01	0.4 ± 0.02	0.84 ± 0.02	0.7 ± 0.01
	Autumn	0.82 ± 0.0004	1.08 ± 0.0005	2.42 ± 0.0001	1.33 ± 0.004	1.40 ± 0.0003	0.46 ± 0.0006	0.67 ± 0.0006	2.01 ± 0.0008	1.13 ± 0.01

DO, T, DIP, and DOP were measured in overlying water of sediments. pH, TOC, TN, TP, OP, HCl-P, NaOH-P, and ALP were measured in sediments. Data are mean ± standard deviation.

### Genetic diversity and phylogenetic tree based on the *phoD* gene

Total DNA was extracted from the microorganisms in sediment samples, and *phoD* gene sequences were acquired by PCR amplification. A total of 881,717 valid sequences were obtained by high-throughput sequencing, with an average length of 362 bp. The number of sequences varied between 6364 and 99,463 at different sampling sites, the average number of sequences being 48,984. The rarefaction curve of all sediment samples tended to be flat, indicating that the sequencing data volume was reasonable at each site (Supplementary Fig. S1). The samples were clustered into 477 OTUs at the 80% similarity threshold^30^. For spring and autumn, the number of shared OTUs was 96; the number of OTUs unique to these two seasons was 238 and 143, respectively. The number of OTUs and the number of valid sequences were larger in autumn than in spring. The number of OTUs for each sample varied between 24 and 152. The distributions of the number of OTUs and the number of valid sequences across the samples are shown in Table 2.

**Table 2:** The richness and diversity of *phoD* genes in sediments of the Sancha Lake.

Season	Sample sites	No. of valid sequences	No. of OTUs	Shannon	Simpson	Chao1	Ace	No. of phyla	No. of classes	No. of orders	No. of families	No. of genera
Spring	L1	10,377	24	2.45	0.6867	25.50	27.09	2	4	6	9	9
	L2	49,225	152	5.96	0.9685	160.33	167.38	5	9	15	19	20
	L3	99,463	47	2.25	0.5717	53.00	55.13	3	7	11	15	17
	L4	6364	54	3.02	0.6670	56.55	59.33	4	6	13	17	20
	L5	7353	44	2.90	0.7754	44.43	46.19	3	5	10	13	14
	L6	94,358	81	4.38	0.9008	84.00	88.24	3	7	13	17	19
	L7	7260	48	3.73	0.8463	48.08	48.94	2	5	9	13	13
	L8	95,250	67	4.09	0.8819	70.24	72.94	3	7	10	13	15
	L9	14,307	41	3.58	0.8469	41.00	41.00	2	4	6	9	10
Autumn	L1	33,806	108	3.63	0.7505	137.29	149.04	6	9	16	17	20
	L2	48,433	127	5.37	0.9461	138.70	146.45	6	8	14	18	19
	L3	52,729	77	4.32	0.9021	89.67	93.99	5	7	8	10	11
	L4	75,516	47	1.95	0.4837	57.11	58.69	4	7	8	10	11
	L5	45,305	85	3.93	0.8596	98.32	104.20	4	7	8	12	13
	L6	62,058	83	3.00	0.6847	99.71	110.19	4	7	9	12	12
	L7	28,879	97	3.55	0.7740	114.65	120.36	3	7	9	11	13
	L8	74,746	68	3.74	0.8510	77.00	83.87	4	8	11	14	16
	L9	76,288	63	4.28	0.9067	65.33	66.73	3	5	8	9	10

Using the statistics of OTUs, we calculated Shannon's index and Simpson's index to represent genetic diversity based on the *phoD* gene and ACE index and Chaol index to represent abundance, as shown in Table 2. The genetic diversity of bacterial communities in the sediments of Sancha Lake was high based on the *phoD* gene. The Shannon’s index varied between 1.95 and 5.96, and the Simpson’s index between 0.4837 and 0.9685. As for abundance, the Chaol index varied between 57.11 and 160.33, and the ACE index between 58.69 and 167.38. The Shannon's index and the Simpson's index were slightly higher in autumn than in spring (P > 0.05). The Chaol index and the ACE index were significantly higher in autumn than in spring (P < 0.05). These results indicated that the *phoD*-harboring bacterial communities in autumn and spring had varying levels of diversity and abundance. In autumn, Shannon's index and Simpson's index were the lowest at the center of the lake and increased in the arm of the lake. The genetic diversity of bacterial communities increased significantly in the tail of the lake.

**Figure 2:**
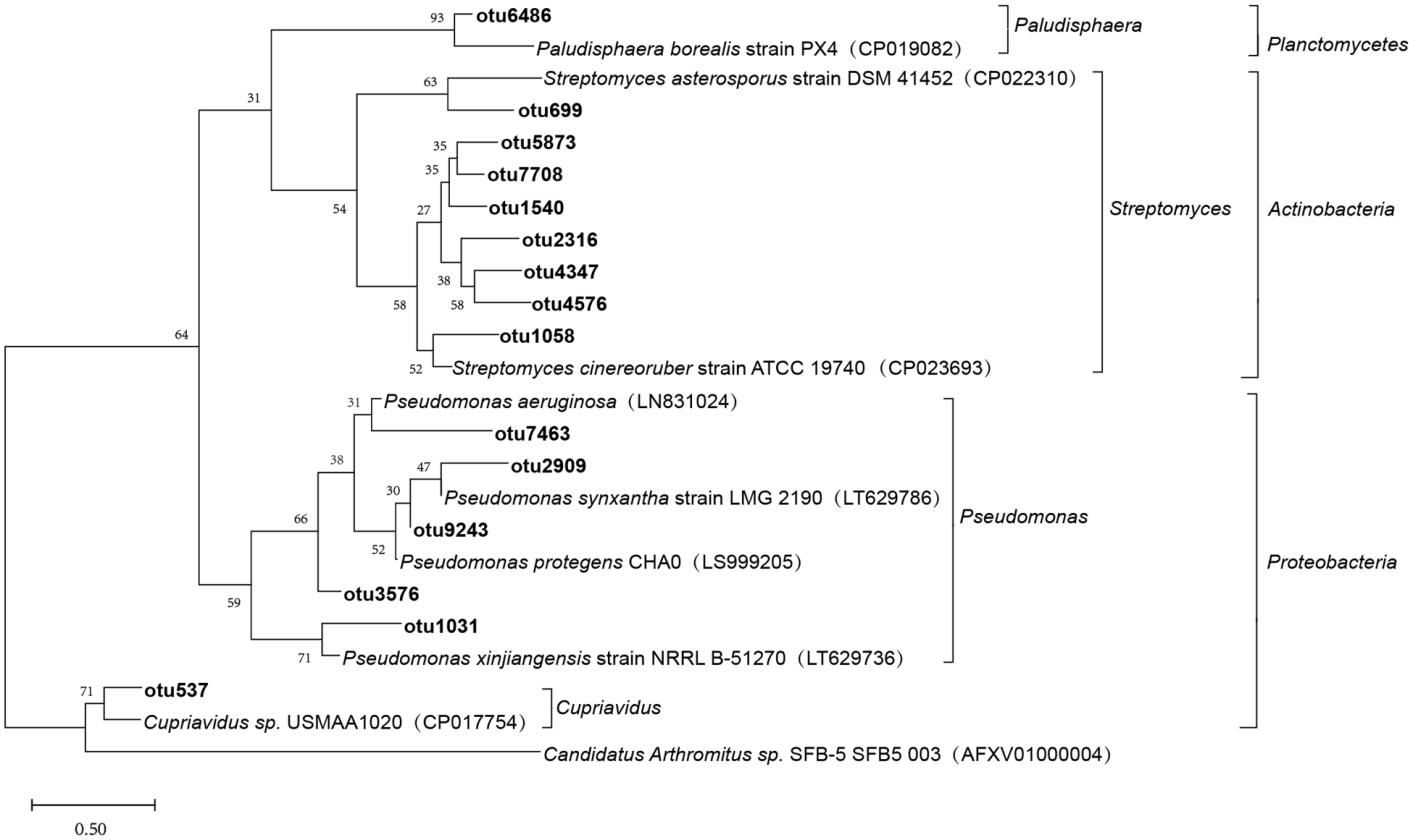
Phylogenetic tree of *PhoD* gene sequences (15 OTUs) from sediments of the Sancha Lake.

A phylogenetic tree was drawn based on representative *phoD* gene sequences for the top 15 OTUs with the highest relative abundance. This phylogenetic tree was composed of three branches (Fig. 2). These sequences were primarily aligned with genera *Pseudomonas* and *Cupriavidus* belonging to the phylum Proteobacteria, genus *Streptomyces* belonging to the phylum Actinobacteria, and genus *Paludisphaer* belonging to the phylum Planctomycetes. Genera *Pseudomonas* and *Streptomyces* were the most observed in all treatments and accounted for 37.97% of all sequences.

### *phoD*-harboring bacterial community composition and structure

**Figure 3:**
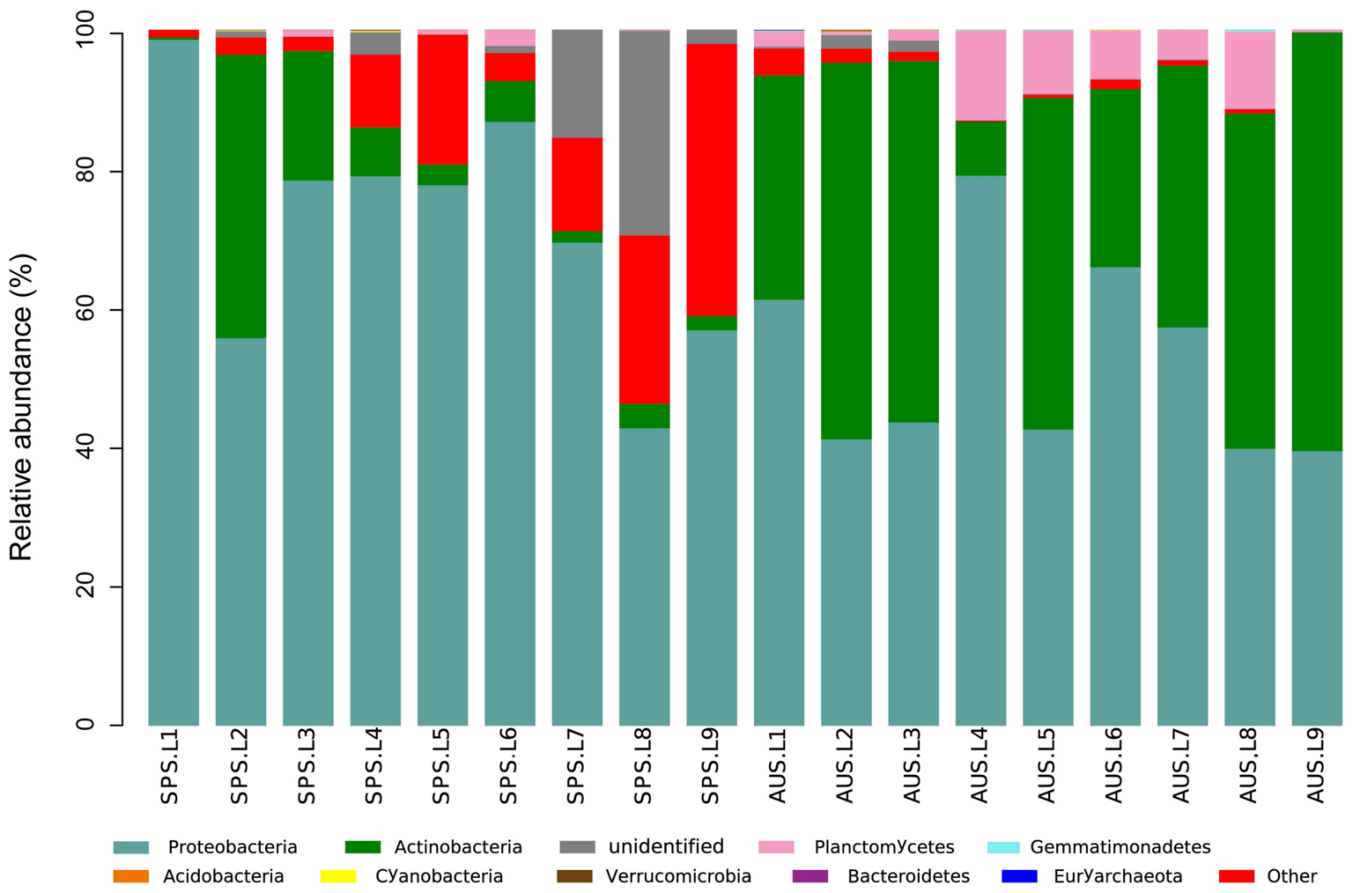
Relative abundance and composition of *phoD*-harboring bacterial phyla detected in the sediments of Sancha Lake in the spring and autumn. *SPS* Spring Location, *AUS* Autumn Location.

In 18 sediment samples, 41 genera belonging to 31 families, 23 orders, 12 classes, and 9 phyla with definite taxonomic information were identified in the *phoD*-harboring bacterial communities. On the phylum level, the distributions and relative abundances of more abundant *phoD*-harboring bacterial communities in the sediments of Sancha Lake in spring and autumn are shown in Fig. 3. Phyla with higher relative abundances (with an average abundance > 1%) were Proteobacteria (with an average abundance of 61.9% and a relative abundance of 39.4–98.6%), Actinobacteria (24.9%, 0.3–60.1%), and Planctomycetes (2.9%, 0.1–13.0%). Among the above, Proteobacteria and Actinobacteria were integral components of *phoD*-harboring bacterial communities. The relative abundance of Proteobacteria in spring was significantly higher than that in autumn (P < 0.05). Besides, the relative abundances of Actinobacteria and Planctomycetes in spring were significantly lower than that in autumn (P < 0.05).

**Figure 4:**
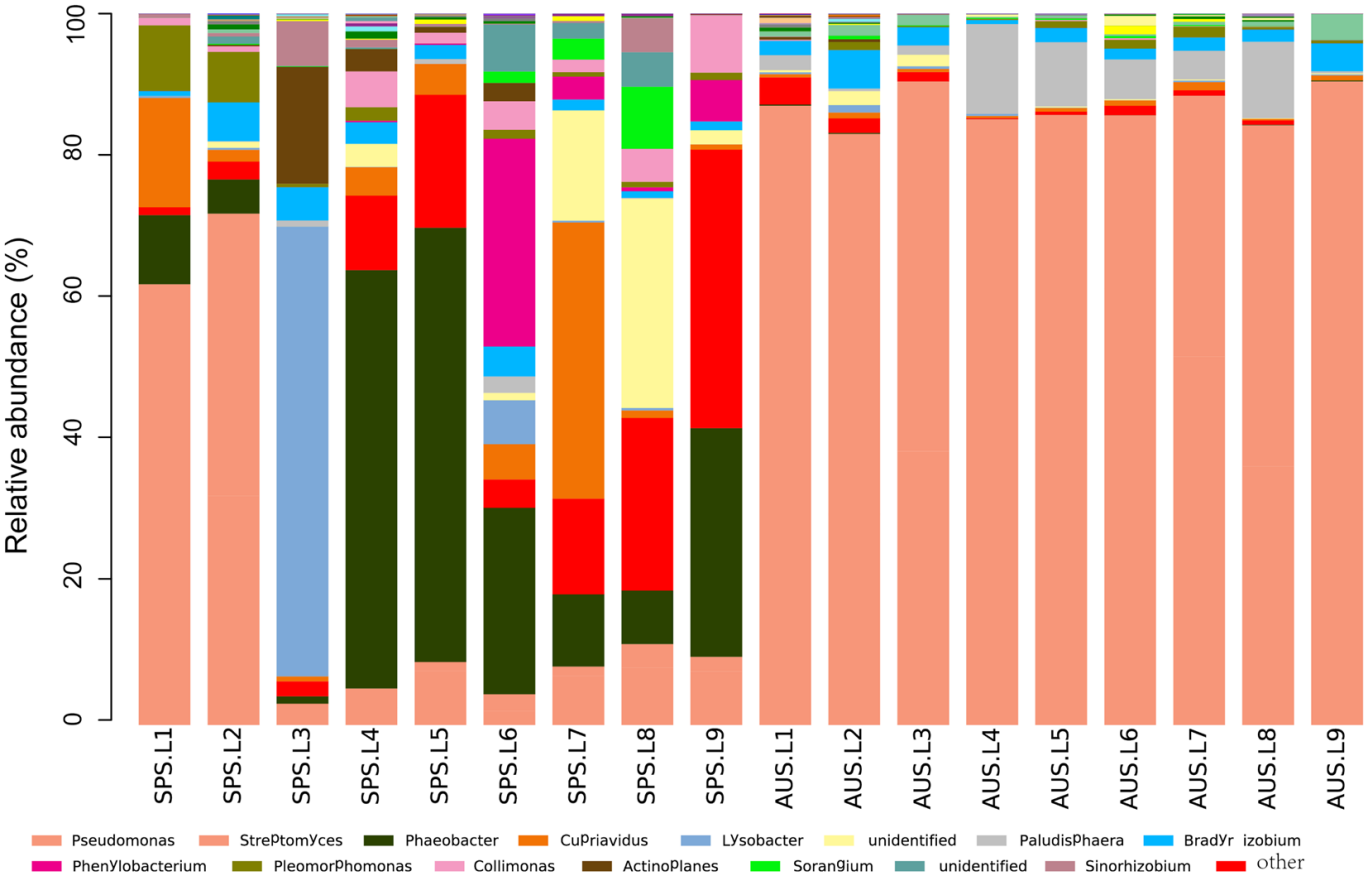
Relative abundance and composition of *phoD*-harboring bacterial genera detected in the sediments of Sancha Lake in the spring and autumn. *SPS* Spring Location, *AUS* Autumn Location.

On the genus level, the distributions and relative abundances of more abundant *phoD*-harboring bacterial communities in the sediments of Sancha Lake in spring and autumn are shown in Fig. 4. Genera with higher relative abundances (average abundance > 1%) were *Pseudomonas* (30.5%, 1.4–77.4%), *Streptomyces* (23.1%, 0.3–60.1%), *Phaeobacter* (11.8%, 0.0–58.8%), *Cupriavidus* (4.3%, 0.2–38.8%), *Lysobacter* (4.1%, 0.0–63.2%), *Paludisphaera* (2.8%, 0.1–10.8%), *Bradyrhizobium* (2.5%, 0.6–4.7%), *Phenylobacterium* (2.2%, 0.0–29.2%), *Pleomorphomonas* (1.6%, 0.0–9.2%), *Collimonas* (1.5%, 0.0–8.0%), and *Actinoplanes* (1.3%, 0.0–16.4%). Genera *Pseudomonas*, *Streptomyces*, *Paludisphaera*, *Cupriavidus*, and *Bradyrhizobium* were shared genera at different sampling points in autumn and spring. The genus *Pseudomonas* had the highest relative abundance, accounting for 49.3% of the phylum Proteobacteria. Bacteria belonging to this genus were important components of the *phoD*-harboring bacterial communities in the sediments of Sancha Lake. The metastats test showed that the relative abundances of genera *Pseudomonas*, *Streptomyces* and *Paludisphaera* were significantly higher in autumn than in spring (P < 0.05). By contrast, the relative abundances of the genera *Cupriavidus* and *Phaeobacter* were significantly higher in spring than in autumn (P < 0.05).

**Figure 5:**
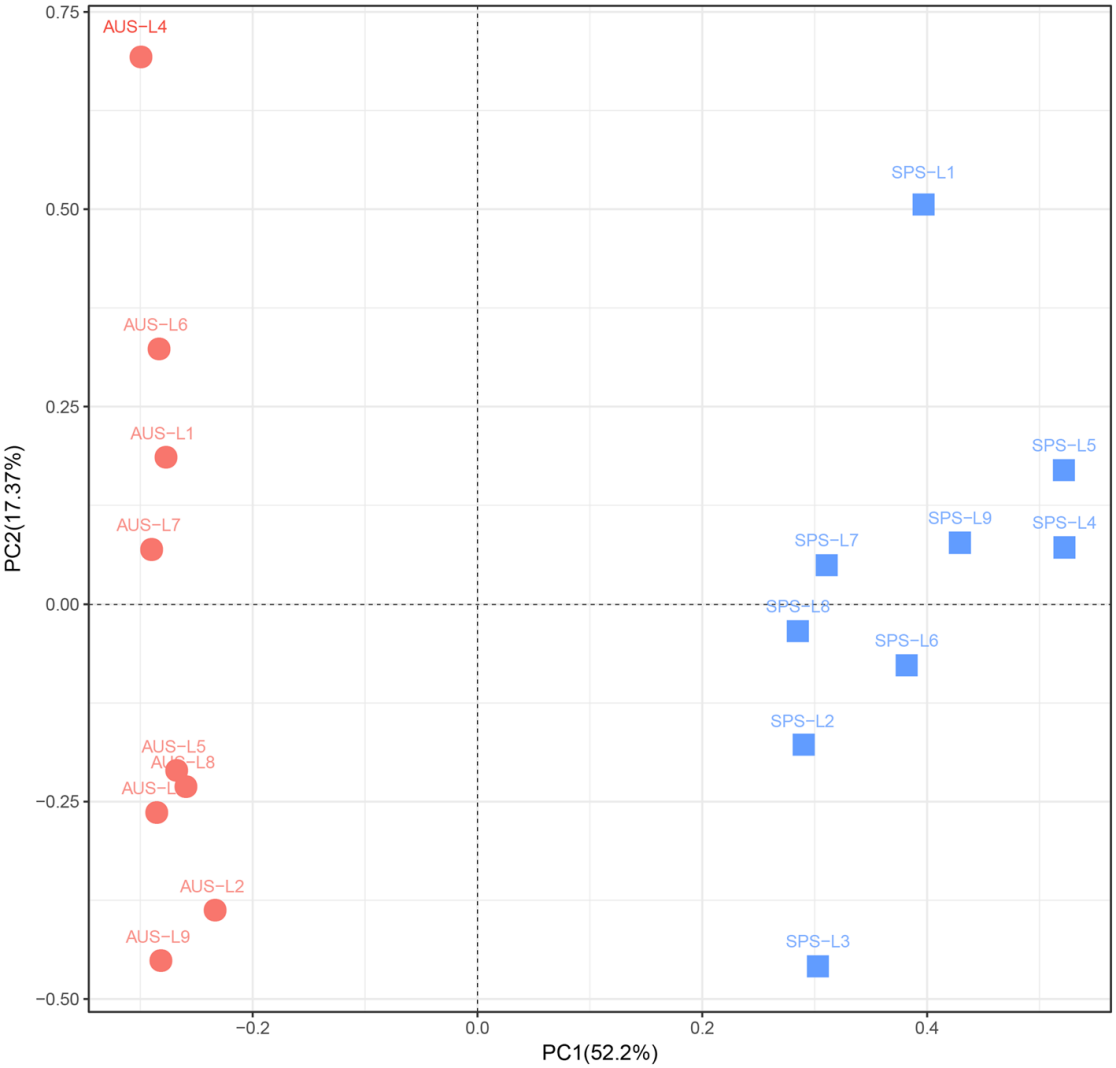
Principal component analysis of *phoD*-harboring bacterial genera detected in the sediments of Sancha Lake in the spring and autumn. *SPS* Spring Location, *AUS* Autumn Location.

The PCA analysis results of the *phoD*-harboring bacterial community structure on the genus level are shown in Fig. 5. PC1 on the x-axis (52.2%) represented the first coordinate axis, along which the samples were differentiated from each other to the maximal degree. PC1 explained 52.2% of all differences in the samples. PC2 on the Y-axis (17.37%) explained 17.37% of all differences in the samples. These two principal components defined a plane, on which over 69.57% of all differences in the samples were shown. The *phoD*-harboring bacterial communities at 9 sampling points in autumn were scatteredly distributed in the first and fourth quadrants. The *phoD*-harboring bacterial communities at 9 sampling points in autumn were concentratedly distributed in the second and third quadrants. On the first coordinate axis with the maximal explained variance ratio, the sampling points represented by circles were effectively differentiated from those by squares. In other words, the intersample differences were primarily attributed to the seasonal factor (spring and autumn) (P < 0.01). Besides, samples from different sites in the same season could be well differentiated along the second coordinate axis. These results indicated that the sampling site had an important impact on the *phoD*-harboring bacterial community structure. The adonis test showed that PCA could differentiate the *phoD*-harboring bacterial communities in autumn and spring with extreme significance (R^2^ = 0.581, P = 0.001).

### Quantification of *phoD* gene abundance

**Figure 6:**
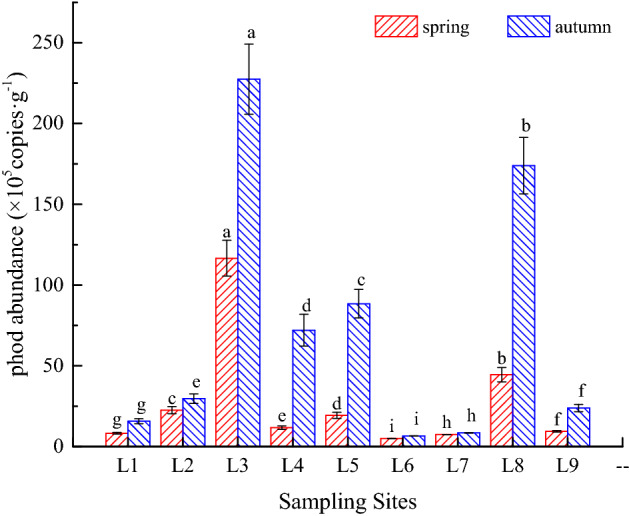
Abundance of *phoD* gene in the sediments of Sancha Lake in the spring and autumn. At the same sampling time, Bars with different letters (a, b, c, d, e, f, g, h, i) at each location point are significantly different at P < 0.05 according to one-way ANOVA. g is dry sediment.

The results of fluorescence quantitative PCR are shown in Fig. 6. The copy number of the *phoD* gene varied between 5.039 × 10^5^ and 2.275 × 10^7^ copies/g (dry sediment) across the sampling points in autumn and spring, the average copy number being 5.065 × 10^6^ copies/g (dry sediment). Significant spatial heterogeneity and seasonal changes were observed in *phoD* gene abundance in sediments of Sancha Lake (0.01 or P < 0.05). The *phoD* gene abundances at different sampling points were significantly higher in autumn than in spring (P < 0.05). In spring, the *phoD* gene abundance was higher in the tail of lake and where cage culture used to be intensive. By contrast, the *phoD* gene abundances were lower where concentrated area of fenced aquaculture used to be intensive and near the regions with intense human activities. It was the lowest in the inflow water area and deep water area of the dam. The sampling points were ranked in a similar order in terms of *phoD* gene abundance in autumn and spring. However, the *phoD* gene abundance fluctuated less significantly in autumn than in spring.

### Correlation between the distribution of *phoD*-harboring bacterial communities in sediments of Sancha Lake and environmental factors

The correlation results between environmental factors vs. diversity and abundance indices and abundance of the bacterial communities based on the *phoD* gene are shown in Table 3. The *phoD* gene abundance had significant or highly significant positive correlations with the pH value, OP and ALP (P < 0.05 or P < 0.01) but significant or highly significant negative correlations with SRP and DO (P < 0.05 or P < 0.01). The number of OTUs clustered based on the *phoD* gene had significant or highly significant positive correlations with T, DOP and TOC (P < 0.05 or P < 0.01), but highly significant negative correlations with OP (P < 0.01). The Shannon's index based on the *phoD* gene had significant positive correlations with the pH value and DOP (P < 0.05), but significant negative correlations with DO, TP and OP (P < 0.05). The Chaol index based on the *phoD* gene had a significant positive correlation with ALP (P < 0.05). Overall, the diversity and abundance indices and abundance of the bacterial communities based on the *phoD* gene were closely related to the physicochemical factors.

**Table 3:** Statistical analysis of diversities and abundances of *phoD* gene with physicochemical parameters.

Diversities	pH	DO	T	DOP	SRP	TOC	TN	TP	OP	HCl-P	NaOH-P	ALP
Abundance	0.636**	− 0.727**	0.177	0.051	− 0.640**	− 0.031	0.163	0.092	0.51*	0.100	0.299	0.843**
OTUs	0.261	− 0.289	0.614**	0.731**	0.031	0.478*	− 0.338	− 0.255	− 0.731**	− 0.290	− 0.066	− 0.068
Shannon	0.637*	− 0.722**	0.350	0.699*	0.124	− 0.411	− 0.380	− 0.649*	− 0.699*	− 0.294	− 0.281	0.024
Simpson	− 0.138	0.052	0.082	− 0.248	0.050	− 0.277	− 0.201	− 0.159	− 0.248	− 0.194	− 0.263	0.627*
ACE	0.390	− 0.419	− 0.204	− 0.071	− 0.074	− 0.257	− 0.427	− 0.214	− 0.071	− 0.261	0.030	− 0.014
Chao1	0.372	− 0.399	0.332	− 0.092	− 0.057	− 0.282	− 0.439	− 0.232	− 0.092	− 0.277	0.007	0.672*

*P < 0.05; **P < 0.01. n = 18.

The results of a linear regression analysis showed that the *phoD* gene copy number had an highly significant positive correlation with the ALP activity (R^2^ = 0.705, y = 0.008X + 0.528, *p* = 0.000). The *phoD* gene abundance had a significant negative correlation with SRP (R^2^ = 0.510, y = − 1644.065X + 93.722, *p* = 0.004). The ALP activity had a significant negative correlation with SRP (R^2^ = 0.631, y = − 16.788X + 1.366, *p* = 0.001).

**Figure 7:**
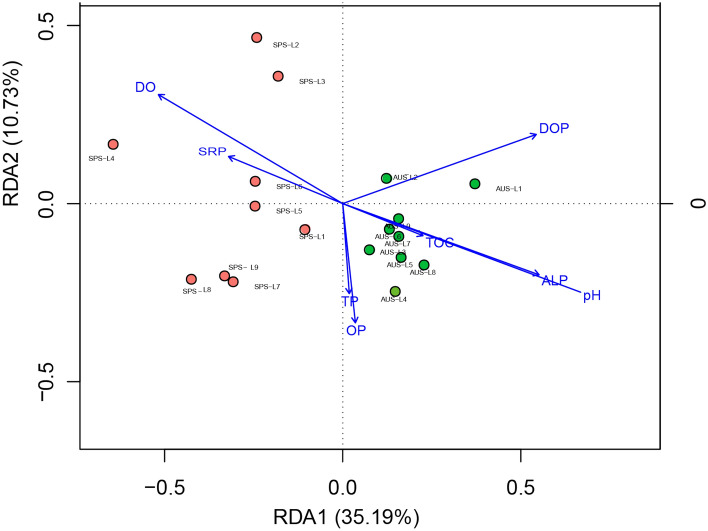
The RDA analysis of *phoD*-harboring bacterial genera and physico-chemical factors in the sediments of Sancha Lake in the spring and autumn. *SPS* Spring Location, *AUS* Autumn Location.

The results of the RDA analysis on the genus level of *phoD*-harboring bacteria are shown in Fig. 7. Dynamic changes in the *phoD*-harboring bacterial communities in sediments of Sancha Lake were influenced by physical and chemical factors in the water bodies and sediments. In autumn and spring, DO, DOP and SRP in overlying water and pH value, TP, OP, ALP and TOC of sediments were environmental factors that had an important impact on the *phoD*-harboring bacterial community structure. The two axes offered an explanatory power of 15.19% and 10.73%, respectively. All samples in autumn fell within the first and fourth quadrants in a concentrated manner; the samples in spring fell within the second and third quadrants in a scattered manner. Thus, the samples in spring and autumn were effectively differentiated from each other (P < 0.05).

## Discussion

### *phoD*-harboring bacterial diversity and community structure

Shifts in the *phoD*-harboring bacteria community were reported recently in many soils and marine. Little is known, however, about *phoD*-harboring bacterial diversity and community structure in eutrophicated sub-deep freshwater lakes. We reported a high diversity of the *phoD* gene in the sediments of Sancha Lake, an eutrophic sub-deep freshwater lake. On the phylum level, Proteobacteria, Actinobacteria and Planctomycetes were integral components of the *phoD*-harboring bacterial communities in the sediments of Sancha Lake. On the genus level, *Pseudomonas*, *Streptomyces*, *Cupriavidus*, and *Collimonas* were dominant bacterial genera in *phoD*-harboring bacterial communities.

Genes encoding functional enzymes have been extensively used as molecular markers to study the diversity, structure and functional activity of microbial communities in ecological science^38^. Bacteria are important mineralizers of organic phosphorus^39^, secreting ALP to mineralize organic phosphorus into orthophosphates that are involved in chemical cycling of phosphorus and maintaining the eutrophication status of lakes^40^. So far, some studies have been conducted using the *phoD* gene as a molecular marker to characterize the genetic diversity and structure of ALP-secreting bacteria in freshwater ecosystems. Zhao et al.^41^ employed RFLP to analyze the diversity of bacterial *phoD* gene in the water bodies of the Pearl River. They reported a high diversity of bacterial *phoD* gene in the Pearl River. *Limnohabitans*, *Plesiocystis* and *Pirellula* are important bacterial genera in the *phoD*-harboring bacterial community. ALP encoded by the *phoD* gene may play an important role in eutrophication of the Pearl River. Valdespino-Castillo et al.^42^ combined high-throughput sequencing with clone library to detect the composition of the *phoD*-harboring bacterial community in shallow Lake Alchichica, Mexico. It was found that Actinobacteria, Alphaproteobacteria, Betaproteobacteria and Gammaproteobacteria were dominant bacterial classes. Zhang et al.^19^ applied fluorescence quantitative PCR and high-throughput sequencing to study the spatial and temporal distribution features of bacterial *phoD* gene in suspended particles in Taihu Lake. It was found that the dominant *phoD*-harboring phylum in all samples was Actinobacteria, followed by Proteobacteria, Cyanobacteria and Gemmatimonadetes. Sun^21^ analyzed the composition and genetic diversity of the *phoD*-harboring bacterial community in the sediments of Lake Chaohu. A high genetic diversity of the *phoD*-harboring bacterial community in sediments was noted, with Actinobacteria, Proteobacteria and Planctomycetes being dominant phyla and *Pseudonocardia* and *Friedmanniella* being dominant genera. The dominant bacterial phyla and genera in the *phoD*-harboring bacterial community in sediments of Sancha Lake were different from those in the Pearl Riverand and in Lake Alchichica, Mexico^41,42^, but similar to those in the sediment of Lake Chaohu and in suspended particles in Taihu Lake^19,21^.

### Relationship between *phoD*-harboring bacterial diversity, abundance and community structure vs. environmental factors

Our study indicated significant correlations between the *phoD*-harboring bacterial community in sediments of Sancha Lake and environmental factors. The *phoD*-harboring bacterial community structure was altered as a response to changes in DO, pH value, TOC and phosphorus content, all of which were environmental factors. The above finding agreed with other published studies. The TN/TP ratio was reported to be a primary environmental factor influencing the *phoD*-harboring bacterial community structure and gene copy number in Lake Alchichica, Mexico^42^. DO, TN and TP were primary environmental factors influencing *phoD* gene abundance of suspended particles in shallow Lake Taihu^19^. The structure and genetic diversity of the *phoD*-harboring bacterial community structure in the sediments of Lake Chaohu were influenced by pH value, DO, T, and TP content^21^. Although the environmental factors influencing the *phoD*-harboring bacterial community vary across the habitats, the phosphorus content seems to have a universal impact on the *phoD*-harboring bacterial community in freshwater ecosystems.

### Response of functional microbes in sediments to available phosphorus in eutrophicated water bodies

The phosphorus content in sediments is closely related to that in overlying water. This is because the overlying water comes into contact with bottom sediments. There are usually material and energy exchanges between the overlying water and bottom sediments due to biological actions^43^. In East Lake, phosphorus is released from sediments due to microbial activities under a low phosphorus concentration in overlying water. The amount of phosphorus released from the sediments was negatively correlated with the phosphorus concentration in the lake water^44^. In Lake Tai, Wang et al.^45^ observed close connections between the changes in phosphorus concentration in overlying water and ALP activity in sediments. Besides, ALP was closely related to the amount of phosphorus-dissolving bacteria in sediments. We calculated Pearson's correlation coefficients and performed ANOVA in the present study. It was found that the bacterial *phoD* gene abundance and ALP activity in the sediments of Sancha Lake were significantly negatively correlated with SRP in overlying water (P < 0.05). However, the *phoD* gene abundance had an highly significant positive correlation with ALP activity (P < 0.01). Therefore, *phoD*-harboring bacteria were the main producers of ALP, which made important contributions to SRP in overlying water.

Algae proliferative massively in eutrophic water bodies and utilize a large amount of phosphorus during their growth, disrupting the balance of phosphorus release from sediments. As a result, the amount of phosphorus released from sediments to water increases. Organic phosphorus is the form of phosphorus first released from sediments in the presence of algae and has a higher bioavailability^46^. In the present study, the *phoD* gene abundance and ALP activity of the bacterial community in sediments of Sancha Lake were significantly higher in autumn than in spring (P < 0.05). Besides, they were closely related to organic phosphorus content in sediments. We inferred that the vigorous growth of aquatic plants in Sancha Lake in autumn significantly increased the diversity of the *phoD* gene in sediments. As the demand of aquatic plants for available phosphorus increased, the *phoD*-harboring bacterial community began to show stronger ability to decompose organic phosphorus, resulting in a greater amount of phosphorus released to overlying water. This explanation was supported by higher relative abundances of *Pseudomonas* and *Streptomyces* in sediments in autumn, as observed in the present study. *Pseudomonas* and *Streptomyces* are among the most important organic phosphorus-degrading bacteria in sediments^47^. The growth of algae and other plankton leads to the "pumping" of phosphorus in sediment^48^. With the continuous reproduction of algae in the Sancha Lake in autumn, the demand for phosphorus gradually increases, and the phosphorus consumption in the overlying water can no longer be supplemented by the independent diffusion alone. ALP mineralizes and hydrolyzes OP in sediments, releasing SRP, which is needed for Alga growth. Therefore, in Sancha Lake, the rapid decline of SRP in overlying waters due to the increasing algaeal photosynthesis in the early growth of algae induces the ALP, promotes the growth of *Pseudomonas* and *Streptomyces* and alleviates the phosphorus restriction.

### Potential limitations of the methodological approach used

The inherent methodological limitation of *phoD* primers does to be acknowledged although it has not limited the importance of this study. The species of *phoD*-harboring bacteria recovered with primer sets ALPS-F730/ALPS-S1101 in this study seem to be restricted to a few bacterial phyla. Nonetheless, our result showed that a significant positive correlation between *phoD* gene abundance and ALP activity (R^2^ = 0.715, *p* < 0.01), indicating that the identified *phoD* species represented the majority of *phoD* populations in our sediment. Newly designed primers based on metagenome databases will probably help to detect larger *phoD* gene diversity in future studies^49^. Moreover, future research could consider the *phoD* expression level, which is a direct indication of sediment ALP activity. Finally, SRP release from fractions has biological, physical and chemical effects, and can be carried out under anaerobic and oxidizing conditions.

We did not investigate differences between the "biotic" and "abiotic" regulation of SRP release from sediments with oxic or anoxic bottom waters. We believes that these aspects need to be further studied in future.

## Conclusions

We observed a high diversity of the *phoD* gene in sediments of Sancha Lake, which implies an abundance of phosphorus-dissolving bacterial resources.

The *phoD*-harboring bacterial community structure in sediments of Sancha Lake showed a significant difference in spring and autumn, but no apparent spatial heterogeneity. Significant spatial heterogeneity and seasonal changes were observed in *phoD* gene abundance.

pH value, DO, TOC, ALP and phosphorus content were important environmental factors influencing genetic diversity and structure of the *phoD*-harboring bacterial community in the sediments of Sancha Lake. The spatial and seasonal changes in the *phoD* abundance and *phoD*-harboring bacterial community structure represent a dynamic response to SRP in overlying water. The *phoD*-harboring bacterial community may play an important role in the transfer and conversion of phosphorus in sediments and overlying water in Sancha Lake, supplementing phosphorus to overlying water.

## Supplementary Information

Supplementary Figure S1.

## Data Availability

The datasets generated during the current study are available in the NCBI Sequence Read Archive (SRA) database repository, [Accession Number PRJNA623151].

## References

[1] Smith VH (2003). Eutrophication of freshwater and coastal marine ecosystems: A global problem. Environ. Sc. Pollut. R. Int..

[2] Jeppesen E, Sondergaard M, Jensen JP (2005). Lake responses to reduced nutrient loading an analysis of contemporary long term data from 35 case studies. Freshw. Biol..

[3] Kim LH, Choi E, Michal KS (2003). Sediment characteristics, phosphorus types and phosphorus release rates between river and lake sediments. Chemosphere.

[4] Jiang XJ, Xiang C, Yao Y (2008). Effects of biological activity, light, temperature and oxygen on phosphorus release processes at the sediment and water interface of Taihu Lake, China. Water Res..

[5] Wang SR, Jin XC, Bu QY (2008). Effects of dissolved oxygen supply level on phosphorus release from lake sediments. Colloids Surf. A.

[6] Miao SY, De-Laune RD, Jug-Sujinda A (2006). Influence of sediment redox conditions on release/solubility of metals and nutrients in a Louisiana Mississippi River deltaic plain freshwater lake. Sci. Total Environ..

[7] Smits JGC, Van Beek JKL (2013). ECO: A generic eutrophication model including comprehensive sediment-water interaction. PLoS ONE.

[8] Topcu A, Pulatsu S (2014). Phosphorus fractions and cycling in the sediment of a shallow eutrophic pond. Tarim Bilim. Derg..

[9] Jeppesen E, Sondergaard M, Jensen JP (2005). Lake responses to reduced nutrient loading-an analysis of contemporary long-term data from 35 case studies. Freshw. Biol..

[10] Song CL, Cao XY, Liu YB (2009). Seasonal variations in chlorophyll a concentrations in relation to potentials of sediment phosphate release by different mechanisms in a large chinese shallow eutrophic lake (Lake Taihu). Geomicrobiol. J..

[11] Pop O, Martin U, Abel C, Müller JP (2002). The twin-arginine signal peptide of *PhoD* and the TatAd/Cd proteins of *Bacillus subtilis* form an autonomous tat translocation system. J. Biol. Chem..

[12] Luo HW, Zhang HM, Long RA (2011). Depth distributions of alkaline phosphatase and phosphonate utilization genes in the North Pacific Subtropical Gyre. Aquat. Microb. Ecol..

[13] Tan H (2012). Long-term phosphorus fertilisation increased the diversity of the total bacterial community and the *phoD* phosphorus mineraliser group in pasture soils. Biol. Fertil. Soils.

[14] Wan WJ (2021). Spatial differences in soil microbial diversity caused by pH-driven organic phosphorus mineralization. Land Degrad. Dev..

[15] Chen X (2017). Response of soil *phoD* phosphatase gene to long-term combined applications of chemical fertilizers and organic materials. Appl. Soil Ecol..

[16] Sagnon A (2022). Amendment with Burkina Faso phosphate rock-enriched composts alters soil chemical properties and microbial structure, and enhances sorghum agronomic performance. Sci. Rep..

[17] Chhabra S (2012). Fertilization management affects the alkaline phosphatase bacterial community in barley rhizosphere soil. Biol. Fertil. Soils.

[18] Luo HW, Benner R, Long RA, Hu JJ (2009). Subcellular localization of marine bacterial alkaline phosphatases. Proc. Natl. Acad. Sci..

[19] Zhang TX (2020). Suspended particles *phoD* alkaline phosphatase gene diversity in large shallow eutrophic Lake Taihu. Sci. Total Environ..

[20] Li H (2021). Nutrients regeneration pathway, release potential, transformation pattern and algal utilization strategies jointly drove cyanobacterial growth and their succession. J. Environ. Sci..

[21] Sun TT, Huang T, Liu YX (2022). Effects of cyanobacterial growth and decline on the *phoD*-harboring bacterial community structure in sediments of Lake Chaohu. J. Lake Sci..

[22] Li Y, Ai MJ, Sun Y, Zhang YQ, Zhang JQ (2017). *Spirosoma lacussanchae* sp. nov., a phosphate-solubilizing bacterium isolated from a freshwater reservoir. Int. J. Syst. Evol. Microbiol..

[23] Li Y, Zhang JJ, Xu WL, Mou ZS (2019). Microbial community structure in the sediments and its relation to environmental factors in eutrophicated Sancha Lake. Int. J. Environ. Res. Public Health.

[24] Jia BY, Tang Y, Fu WL (2013). Relationship among sediment characteristics, eutrophication process and human activities in the Sancha Lake. China Environ. Sci..

[25] Li Y, Zhang JJ, Zhang JQ, Xu WL, Mou ZS (2019). Characteristics of inorganic phosphate-solubilizing bacteria from the sediments of a Eutrophic Lake. Int. J. Environ. Res. Public Health.

[26] Ruban V, Brigault S, Demare D, Philippe AM (1999). An investigation of the origin and mobility of phosphorus in freshwater sediments from Bort-Les-Orgues reservoir, France. J. Environ. Monit..

[27] Ruban V, López-Sánchez JF, Pardo P (2001). Harmonized protocol and certified reference material for the determination of extractable contents of phosphorus in freshwater sediments: A synthesis of recent works. Fresenius J. Anal. Chem..

[28] Li Y, Zhang JQ, Gong ZL, Fu WL, Wu DM (2019). Fractions and temporal and spatial distribution of phosphorus in the sediments of Sancha lake. Appl. Ecol. Environ. Res..

[29] Li Y, Zhang JQ, Gong ZL, Xu WL, Mou ZS (2019). Gcd gene diversity of quinoprotein glucose dehydrogenase in the sediment of Sancha lake and its response to the environment. Int. J. Environ. Res. Public Health.

[30] Luo GW (2017). Long-term fertilisation regimes affect the composition of the alkaline phosphomonoesterase encoding microbial community of a vertisol and its derivative soil fractions. Biol. Fertil. Soils.

[31] Lagos L (2016). Effect of phosphorus addition on total and alkaline phosphomonoesterase-harboring bacterial populations in ryegrass rhizosphere microsites. Biol. Fertil. Soils.

[32] Acuña J (2016). Bacterial alkaline phosphomono-esterase in the rhizospheres of plants grown in chilean extreme environments. Biol. Fertil. Soils.

[33] Nicholas AB (2013). Quality-filtering vastly improves diversity estimates from Illumina amplicon sequencing. Nat. Methods..

[34] Price MN, Dehal PS, Arkin AP (2009). FastTree: Computing large minimum evolution trees with profiles instead of a distance matrix. Mol. Biol. Evol..

[35] Fan XF, Xing P (2016). The vertical distribution of sediment archaeal community in the (black bloom) disturbing Zhushan Bay of Lake Taihu. Archaea.

[36] White JR, Nagarajan N, Pop MO (2009). Statistical methods for detecting differentially abundant features in clinical metagenomic samples (differential abundance in clinical metagenomics). PLoS Comput. Biol..

[37] Hu H, Chen XJ, Hou FJ, Wu YP, Cheng YX (2017). Bacterial and fungal community structures in loess plateau grasslands with different grazing intensities. Front. Microbiol..

[38] Dai JY (2016). Bacterial alkaline phosphatases and affiliated encoding genes in natural waters: A review. J. Lake Sci..

[39] Chróst RJ, Overbeck J (1987). Kinetics of alkaline phosphatase activity and phosphorus availability for phytoplankton and bacterio-plankton in lake plusee (North German Eutrophic Lake). Microb. Ecol..

[40] Margalef O (2017). Global patterns of phosphatase activity in natural soils. Sci. Rep..

[41] Zhao DD, Luo JF, Huang XY, Lin WT (2015). Diversity of bacterial APase *phoD* gene in the Pearl River water. Acta Sci. Circum..

[42] Valdespino-Castillo PM (2014). Alkaline phosphatases in microbialites and bacterioplankton from Alchichica soda lake, Mexico. FEMS Microbiol. Ecol..

[43] Ni ZK, Li Y, Wang SR (2022). Cognizing and characterizing the organic phosphorus in lake sediments: Advances and challenges. Water Res..

[44] Han SS, Wen TM (2004). Phosphorus release and affecting factors in the sediments of eutrophic water. J. Ecol..

[45] Wang FF, Qu JH, Hu YS (2012). Spatio-temporal characteristics and correlation of phosphate, pH and alkaline phosphatase on water-sediment interface of Lake Taihu. Ecol. Environ. Sci..

[46] Lu YM (2020). Bioavailability of organic phosphorus in Lake Chaohu sediments. J. Environ. Eng. Technol..

[47] LeBrun ES, King RS, Back JA, Kang S (2018). Microbial community structure and function decoupling across a phosphorus gradient in streams. Microb. Ecol..

[48] Zhang J (2023). Connecting sources, fractions and algal availability of sediment phosphorus in shallow lakes: An approach to the criteria for sediment phosphorus concentrations. J. Environ. Sci..

[49] Hu YJ (2018). Effects of long-term fertilization on *phoD*-harboring bacterial community in Karst soils. Sci. Total Environ..

