# Supplementary material for: The spatio-temporal distribution of alkaline phosphatase activity and phoD gene abundance and diversity in sediment of Sancha Lake
Source: Sci Rep. 2023 Feb 22;13:3121. doi: 10.1038/s41598-023-29983-1 (PMC9946943; doi:10.1038/s41598-023-29983-1)
Supplement: Supplementary file 1 — Supplementary Figure S1. [file 41598_2023_29983_MOESM1_ESM.pdf]

The Spatio-Temporal Distribution of Alkaline Phosphatase Activity and *phoD* Gene Abundance and Diversity in Sediment of Sancha Lake

Yong Li [1,*], Xintao Yu [1], Huan Liu [1] and Zhilian Gong [2,*]

[1]Faculty of Geosciences and Environmental Engineering, Southwest Jiaotong University, Chengdu 610059, China

[2]School of Food and Biological Engineering, Xihua University, Chengdu 610039, China

**Correspondence:Yong Li，liyong@swjtu.edu.cn，Faculty of Geosciences and Environmental Engineering, Southwest Jiaotong University, Chengdu 610059, China.*

Zhilian Gong, 0120020092@mail.xhu.edu.cn,School of Food and Biological Engineering, Xihua University, Chengdu 610039, China

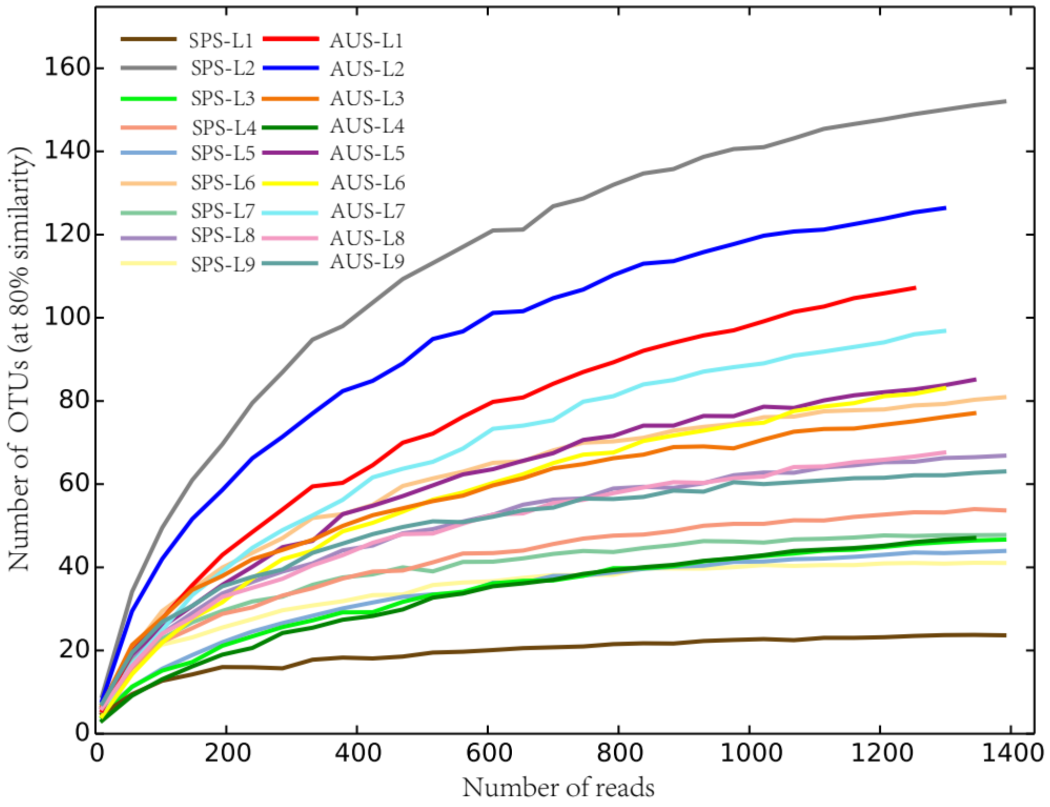

Note:SPS:spring; AUS: autumn.

Supplementary Fig. S1. Rarefaction curves of PhoD gene sequences for the sediment samples of the Sancha Lake in spring and in autumn.
